# Rate of improvement of CF life expectancy exceeds that of general population—Observational death registration study^☆^^☆☆^

**DOI:** 10.1016/j.jcf.2013.12.002

**Published:** 2014-07

**Authors:** Matthew N. Hurley, Tricia M. McKeever, Andrew P. Prayle, Andrew W. Fogarty, Alan R. Smyth

**Affiliations:** a Division of Child Health, Obstetrics and Gynaecology, School of Medicine, University of Nottingham, E Floor East Block, Queens Medical Centre, Nottingham, NG7 2UH, United Kingdom; b Division of Epidemiology and Public Health, School of Medicine, Nottingham City Hospital, Hucknall Road, Nottingham, NG5 1PB, United Kingdom

**Keywords:** Cystic fibrosis, Survival, Mortality, Healthcare

## Abstract

**Background:**

It is unclear why cystic fibrosis (CF) survival has improved. We wished to quantify increases in CF median age of death in the context of general population survival improvement.

**Method:**

Death registration data analysis (US, England & Wales (E&W)—1972–2009).

**Results:**

CF median age of death is higher in US than E&W and greater for males, opposite to that of death from all causes. CF median age of death has increased by 0.543 life years per year (E&W, US combined (95% confidence interval 0.506, 0.582)). The difference in median age at death between those dying from all causes and CF decreased in both territories. CF median age of death for males is greater than for females in both territories. This gap has not narrowed.

**Conclusion:**

The median age of death of people with CF is improving more rapidly than that of the general population in US and E&W.

## 1 Introduction

Cystic fibrosis (CF) is the commonest autosomal recessive condition among Caucasians and affects over 9000 people in the UK [1], over 30 000 in the US [2] and 70 000 people worldwide [3]. Those affected suffer from the direct results of mutations in the *CFTR* gene, causing CFTR dysfunction. This leads to increased viscosity of their secretions leading, in many cases, to recurrent lung infections, pancreatic insufficiency and diabetes mellitus. These factors are associated with a reduced life expectancy. In successive cohorts, survival, lung function and BMI have all been demonstrated to improve over the last twenty years [2], [4], [5]. The median life expectancy for a child born in the UK in the year 2000 was estimated to be greater than 50 years [4].

Although recent decades have seen a great improvement in survival of individuals with CF, the explanation for this improvement is not clear. Instead of observing a step-wise change associated with each new intervention, a continuous increase in the age at death over time is evident [4].

As factors responsible for influencing survival in CF are intricately linked to survival in the general population, we wished to explore the effect of the advances in CF care by quantifying any improvement in median age of death of those with CF in excess of that observed in the general population.

## 2 Method

Mortality data for each year by cause of death (all-cause and CF deaths) and sex were retrieved from the Office of National Statistics for England & Wales (E&W) (1968–2009) and Centre of Disease Control (CDC: WONDER) for United States (1979–2009). For the England & Wales data, over the age of 1 year data are available in 4-year bands whereas the US data above the age of 1 is grouped in 4-year age bands until the age of 25 years and subsequently the bands consist of 10-year age bands. Median age at death was interpolated for data from each country for all-cause mortality and CF mortality by sex. Data use has been restricted to 1972 onward as prior to this date the CF median age of death in England & Wales was less than one year of age and so interpolation of a median age of death was not possible. Median age of death has been used in this study instead of survival as the latter, in estimating the survival chances of individuals, relies upon a cohort methodology which in this case would be unfeasible as it would involve retrieving death certificates of millions of people.

Data were imported into StataSE 12 (StataCorp LP). The absolute difference between and within groups (cause of death, sex and country) over time was calculated. By constructing a linear regression model of the differences between median age of death for all-causes and CF, it is possible to determine gains made for those with CF against a background of increasing life expectancy for the general population.

Sensitivity analyses were performed using datasets covering reduced timespans (1984–2004), to ensure that the trends observed were consistent across the dataset and not disproportionately driven by a single year.

A subgroup of those that died in childhood between the ages 1 to 19 years in England & Wales was used to determine trends in childhood annual age of death compared to other causes of childhood mortality. A linear regression model of the difference between childhood all-cause and CF mortality was constructed to examine trends over time.

Ethical approval was not required.

## 3 Results

The death registration data detailed almost 86 million deaths for the two territories combined, of which 17,861 entries were for those who died with CF (Table 1).

**Table 1:** Numbers of deaths recorded for general population and cystic fibrosis (CF) stratified by territory and sex. US data 1979–2009; England & Wales data 1972–2009.

	England & Wales	US	Total
*General population*			
Males	10 414 879	33 000 866	43 415 745
Females	10 857 458	31 665 675	42 523 133
Total	21 272 337	64 666 541	85 938 878

*CF*			
Males	2247	6556	8803
Females	2375	6683	9058
Total	4622	13 239	17 861

### 3.1 Age of death from all causes in England & Wales is greater than that in the US and age of death of women is greater than that of men

**Fig. 1:**
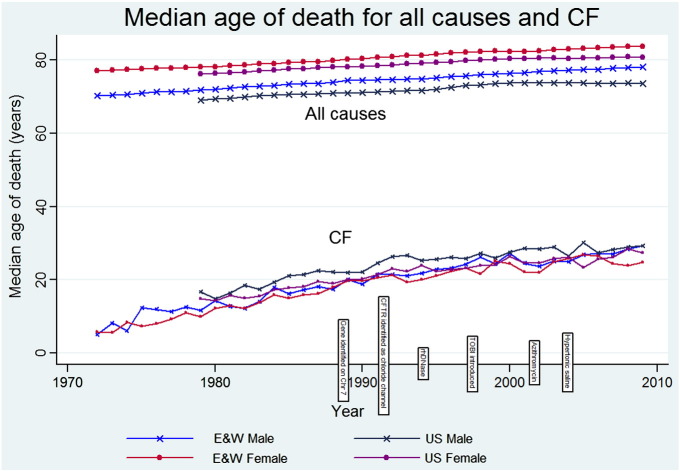
Median age of death in England & Wales and the US for CF and all-cause mortality with milestones in care [29], [30].

Fig. 1 illustrates the trend of continual improvement in the age at death for all-cause mortality over time, increasing by 0.202 life years per year (95% confidence interval 0.135, 0.270) for England & Wales and by 0.165 life years per year (95% confidence interval 0.064, 0.266) for USA combined from 1972–2009. It is also clear that age at death in England & Wales for all-cause mortality is consistently higher than that in the US. There is also a persisting sex difference favouring a greater female age at death compared to males in both England & Wales and the US.

### 3.2 Age of death from CF is greater in the US compared to the UK and the age of death of men is greater than that of women in both countries

Increasing median age of death from CF is demonstrated over time in both countries as a group increasing by 0.543 life years per year (95% confidence interval 0.506, 0.582). Median age at death with a diagnosis of CF is greater in the US than in England & Wales for all years data were available. In 2009, the median age of death in the USA was 23.449 (Interquartile range 16.379, 27.603) and in England & Wales it was 22.125 (IQR—16.602, 31.992).

Similar to death from all causes, a sex difference is also evident in the CF series however, in this instance favouring a greater age at death for males in both countries (p > 0.0001) (Fig. 1).

### 3.3 Improvements in the age of death from CF exceed that of the improvement observed for the general population in the US

The absolute difference in the annual age of death from CF in the USA compared to all-cause mortality from 1979 to 2009 decreased from 52.34 to 44.11 years for males (p < 0.001) and from 61.22 to 53.2 years for females (p < 0.001). This represents a 2.86 and 2.77 fold improvement in age at death for CF compared to all-cause mortality (males and females respectively). The reduction in the difference between age at death between CF and all causes for males equates to 0.276 person years per year (95% confidence interval − 0.336, − 0.217) and for females 0.268 person years per year (95% confidence interval − 0.312, − 0.224) (Table 2).

**Table 2:** Regression model of the difference in annual age of death (rate of change in life years per year) observed between all-causes and CF in England and Wales (E&W) and USA. Negative values indicate a narrowing of the difference between the groups under test.

Comparison	Difference in annual age of death (life years per year)	95% confidence interval		p-Value
*CF*				
US vs E&W males	− 0.095	− 0.158	− 0.032	0.005
US vs E&W females	− 0.059	− 0.126	0.008	0.083

*E&W*				
CF males vs. females	0.018	− 0.034	0.069	0.494
CF vs all cause males	− 0.365	− 0.412	− 0.319	< 0.001
CF vs all cause females	− 0.367	− 0.417	− 0.317	< 0.001

*USA*				
CF males vs. females	0.013	− 0.043	0.070	0.628
CF vs all cause males	− 0.276	− 0.336	− 0.217	< 0.001
CF vs all cause females	− 0.268	− 0.312	− 0.224	< 0.001

### 3.4 Improvements in the age of death from CF exceed that of the improvement observed for the general population in England & Wales

Over the same time period for which data is available in the US (1979–2009) the absolute difference in the annual age of death from CF in England and Wales compared to all causes reduced from 60.09 to 50.61 for males (p < 0.001) and 68.07 to 59.04 for females (p < 0.001). This represents a 2.35 and 2.62 fold improvement in age at death for CF (vs. the general population) in England & Wales over the period for which we have US data, and a 3.12 and 2.82 fold improvement over the whole time period for which we have data for England & Wales (males and females respectively). The reduction in the difference between annual age of death of CF and all causes equates to 0.365 person years per year (95% confidence interval − 0.412, − 0.319) for males and 0.367 person years per year (95% confidence interval − 0.417, − 0.317) for females (Table 2).

### 3.5 The differences in age of death from CF in England & Wales and the US is narrowing

The difference in annual age of death between the two countries for both sexes appears to be reducing over time, although this only reaches statistical significance for males. The difference between the US and England & Wales for males is reducing at a rate of 0.095 person years per year (p = 0.005) and for females a rate of 0.059 person years per year (p = 0.083) (Table 2).

### 3.6 The sex difference in age of death from CF in England & Wales and the US appears to be static

In the context of improvements in CF annual age of death and a reduction in the differences between countries over time, the sex difference in age at death however persists. There is no significant reduction in the difference between males and females observed in either England & Wales (0.018 person years per year, p = 0.494) or the US (0.013 person years per year, p = 0.628).

These observations were not qualitatively changed using sensitivity analyses of varying time periods.

### 3.7 Stratified childhood analysis

**Fig. 2:**
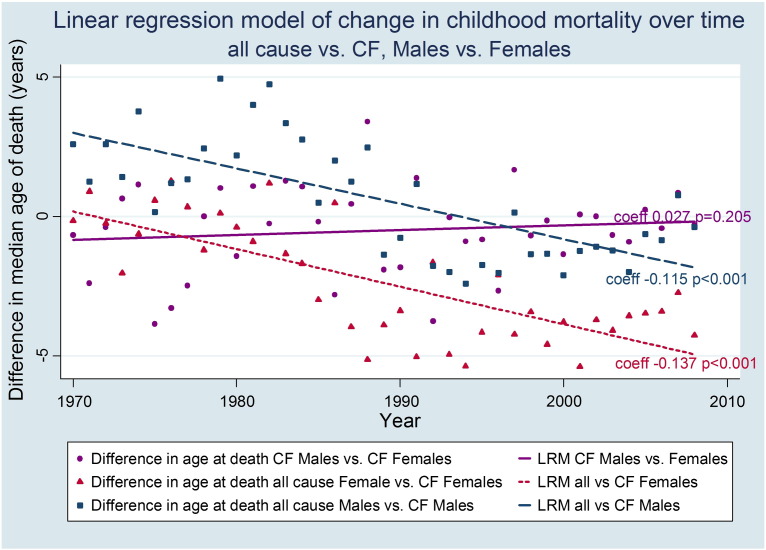
Linear regression model of differences between CF and all-cause childhood (1–19 years) mortality.

Similar patterns of improvement are observed when limiting the analysis to those who do not survive childhood (ages 1–19 years) in England & Wales. (Fig. 2). While the differences in age at death between CF and all causes for males and females have not only reduced but also improved beyond that seen for children dying of other causes, the differences between males and females are unchanged (p = 0.205)

## 4 Discussion

We aimed to quantify the improvement in annual age of death in CF by comparing this improvement with that observed for the general population's all-cause mortality in England & Wales and the US. We have documented a significant improvement in annual age of death for those with CF in excess of that observed for all-cause mortality in both countries. While annual age of death in the general population is greater in England & Wales compared to the US, CF annual age of death appears to be greater in the US, although this difference appears to be narrowing.

There remains evidence of a sex-specific disparity favouring female annual age of death in the general population and male annual age of death in those with CF. In contrast to differences between country and CF/all-cause mortality, the sex difference does not appear to be reducing over time.

This is the first study to quantify improvements in annual age of death of CF in relation to all-cause mortality. While the deaths from CF are nested in those of the general population(s), the percentage is so small (UK for 2009—CF/all deaths—;0.02%) that the CF death rate does not significantly contribute to that from all causes.

It has previously been established that sex combined mortality rates in the US are higher than those in the UK, and using gross domestic product health expenditure (GDPHE) the US has been less efficient and effective in reducing mortality compared to the UK [6]. This may explain the survival advantage of the general population in England & Wales that we observed, however it is unclear why those with CF may have an improved survival in the US compared to those in England & Wales. A similar survival advantage favouring the US had previously been observed in the Netherlands, although in later cohorts (1990–1994) this had disappeared [7].

CF-related survival differences between countries are difficult to explain and have been variably ascribed to differences in rates of care in specialised centres [7] (with centralised care said to confer an advantage [8]), distribution of CF genotypes [9], antibiotic regimens to treat or prevent infection and variations in nutrition and environmental exposure [10]. Countries also vary in rates of diagnosis [11] and it is also clear that the prevalence of CF has reduced since the introduction of newborn screening [12], the implementation of which has varied between countries.

In terms of survival advantage of the general population, social determinants of health appear to be overriding [13]. It is becoming increasingly understood that specifically for CF as well, general factors including household income [14], socioeconomic status [15], body mass index [16], [17] and complications of CF that effect the general population to some degree such as diabetes [18] are predictive of outcome. It is possible that the significantly increased rate of improvement in median age of death of those with CF is because those with CF are more sensitive to these societal factors that include standards of living.

While the influence of sex has been questioned[19], we continue to document a reduced median age of death for women with CF. Women with CF are more likely to be underweight than their male counterparts [20] and poor nutrition is linked to poor prognosis[21]. Furthermore, sex may be associated with more intractable pulmonary infection. Oestrogen induces the mucoid form of *Pseudomonas aeruginosa* (a phenotype associated with poor prognosis [22]), and more mucoid *P. aeruginosa* is isolated from women who experience more pulmonary exacerbations [23].

This study has used ‘current survival’ to describe trends in estimated survival over time. While this method cannot describe trends in terms of birth cohort, and so cannot be predictive of an individuals' life chances. Unfortunately a cohort analysis is unfeasible as this would involve retrieving death certificates of millions of people. The limitations of using current survival however are dwarfed by the strengths of this approach in that we have been able to describe relational trends in survival for such a substantial number of people over a considerable time period.

Patient group specific registries are a relatively recent phenomenon and so there may be a significant lag before sufficient numbers contribute to a survival analysis for future extrapolation to be meaningful. In the meantime, death registry data affords us a means to examine trends in causes of death within a population. Death registry data has the advantage of capturing a vast proportion of deaths, but there may be diagnosis-related biases introduced in that historically deaths due to CF may be overlooked, especially if the cause of death was unrelated to cystic fibrosis, e.g. road traffic collisions. This could conceivably be most marked in those diagnosed with CF in adulthood who may have milder phenotypic presentations of CF. Differences between the US and UK in the coding of death certificates, particularly with regard to cystic fibrosis may introduce bias, for example it may be that male adults investigated for infertility and found to have congenital bilateral absence of the vas deferens (CBAVD) are differentially classed as having cystic fibrosis [24]. The effect of these cases on our study is likely to be very small as are the numbers of cases involved.

It is well recognised that registry data are not entirely representative of the population under study and rely on patient consent, care in a CF centre and accurate reporting to the registry. In one study only 45.9% of patients who died over the age of 45 years in a 14 year time period were reported by the CFF Registry [25]. Our dataset similarly suffers by the risk of misreporting, namely that should a person with CF die of another cause other than CF, then this may be coded by the primary cause of death and not CF. In addition, we have used the median age of death which may mask subtle effects in the distribution of the age of death—for example if changes at one end of the spectrum (such as the introduction of a new medication) were offset by contrasting changes at the other (an increase in death rate). The likelihood of this occurring however is very small, but important to acknowledge.

Previously, while it has been clear that CF survival has improved, the cause of that improvement was unclear, especially in the absence of treatment aimed at ameliorating the molecular defect. It may be suggested however that, as the survival of the general population has also increased over the same time period, the same factors may have contributed to the improvements observed in both contexts—namely improvements in the standard of living and other widespread determinants of health. This study has quantified the cumulative successional effect of a series of improvements in the care of people with CF, improvements in survival in excess of that observed for the general population. It is important to consider reasons why survival of those with CF in the US is better than that in England & Wales, and importantly how the currently static sex difference of those with CF may be remedied.

Until now, no therapy had been available which corrected the molecular defect [4], [26] and disease specific factors such as severity of CFTR dysfunction may not be significantly associated with long term survival [27]. However, the first therapy to potentiate CFTR has just completed phase 3 trials, seems to be highly effective [28] and to date has been introduced in the UK and US. Long term studies will be required to examine the survival advantage that this, and other innovative interventions, may confer.

## 5 Conclusion

Overall, the improvement in survival witnessed among those with CF in absolute terms, in those countries studied, is considerable and supports the assertion that a median survival in excess of 50 years for those born in 2000 should be expected [4].

## Contributions

MH conceived, designed the study and undertook the primary analysis. MH, TM, AP, AWF & ARS contributed to data analysis. All authors were responsible for interpretation of the findings. MH drafted the manuscript, which was critically revised for important intellectual content by all authors. All authors had access to the study data and have agreed the final manuscript for publication. ARS acts as guarantor.
